# Liver Storage Disease in Iran: A Ten Year Study of Liver Biopsies in Children Medical Center Hospital in Tehran-Iran

**DOI:** 10.5812/kowsar.1735143X.718

**Published:** 2011-08-01

**Authors:** Farzaneh Motamed, Maryam Monajemzadeh, Soroush Seifirad, Mandana Ashrafi, Abbas Rasti, Fatemeh Mahjoub

**Affiliations:** 1 Department of Pediatrics Gastroenterology, Children Medical Center Hospital, Tehran University of Medical Sciences, Tehran, IR Iran; 2 Department of Pathology, Children Medical Center Hospital, Tehran University of Medical Sciences, Tehran, IR Iran; 3 Endocrinology and Metabolism Research Center (EMRC), Shariati Hospital, Tehran University of Medical Science, Tehran, IR Iran

**Keywords:** Epidemiology, Gaucher disease, Lysosomal storage diseases, Mucopolysaccharidoses, Glycogen storage disease

## Abstract

**Background:**

Liver storage diseases are rare biochemical and inherited diseases that affect multiorgan systems.

**Objectives:**

This study was performed to determine the rate of storage diseases and their types in liver pathology specimens of subjects who were referred to a tertiary pediatric center.

**Patients and Methods:**

Two pathologists evaluated 2216 pathology specimens (stained with hematoxylin and eosin and periodic acid-Schiff) from subjects who were referred to the largest pediatric tertiary referral center in Iran between 1996 and 2007. Baseline data and clinical and laboratory manifestations were retrieved from the patients' files.

**Results:**

We identified 117 patients who had storage diseases. A combination of clinical and laboratory findings was used to assess the final diagnosis. Glycogen storage disease (GSD) was observed in 85 of cases, compared with lysosomal storage diseases (LSD) in 31 patients and mucopolysaccharidoses in 1 case. LSD was more prevalent in those aged between 1 month and 1 year, whereas GSD was more frequent in those aged between 1 and 6 years. Most of the patients aged between 1 and 6 years. Most patients with LSD and GSD had unknown types of the disease. The most common known types in the LSD and GSD groups were Niemann-Pick disease and GSD type I respectively. The most common clinical and laboratory manifestation was hepatomegaly and abnormal liver enzymes, respectively.

**Conclusions:**

Most of our patients with storage diseases had Gaucher disease. Hepatomegaly and elevated transaminase levels were the most striking finding. However, with regard to the limitations of our methodology, further studies that collect more accurate data are warranted.

## 1. Background

Liver storage diseases are rare biochemical and inherited diseases that affect multiorgan systems. These diseases include lysosomal storage diseases (LSDs), mucopolysaccharidoses (MPS), and glycogen storage disease (GSD) [1][2][3][4][5][6]. LSDs comprise at least 40 diseases that are caused by intralysosomal accumulation of compounds in a variety of cells. The prevalence of LSDs is 1 person per 5000 to 7000 in the Netherlands [7] and 12.25 per 100,000 in the Czech Republic; lipidoses (55%) and MPSs (25%) are the most frequent. Gaucher disease has the highest prevalence among all types-1.13 per 100,000 [8]. The highest occurrence of LSDs was reported in the north of Portugal [9]. LSDs include diseases, such as neuronal ceroid lipofuscinoses (NCL), sphingolipidoses (Gaucher disease, Niemann-Pick syndrome type A,B,C,..), mucopolysaccharidoses (I,II,...,VII), mucolipidoses (II,III,IV), oligosaccharidoses, silalidoses, and GSDII (Pompe disease).

Most of these diseases are inherited as autosomal recessive traits, except MPSII (Hunter) and Fabry disease, which are X-linked [8]. As mentioned above, MPSs are a group of disorders with specific lysosomal enzyme deficiencies that are caused by excess accumulation of glycosaminoglycans. They have a wide spectrum of clinical presentations, due to the abundance of GAGs in connective tissues. Six types of MPS are known, the most severe of which is MPS I. In a Czech study, MPS III had the highest prevalence of all MPSs [6]. GSDs are caused by abnormalities in enzymes that organize the synthesis or degradation of glycogen. Type I is subdivided into Ia and Ib, and type III comprises the IIIa and IIIb subtypes; each of them has a deficiency in glycogen metabolism [6]. The approximate frequency of GSDs is 1 in 20,000 live births, and type I is most common in childhood. All are inherited as autosomal recessive disorders, except for phosphoglycerate kinase deficiency and one form of phosphorylase kinase deficiency, which are X-linked disorders. Hypoglycemia is a common primary manifestation in most of GSDs [1][10]. Overall, the clinical and laboratory features of liver storage diseases are hypoglycemia, physical and mental retardation, changes in blood lactate, triglycerides, cholesterol , transaminases and uric acid, muscular problem, jaundice, glaucoma, neurologic, renal, or muscular degeneration, unexplained hepatomegaly, splenomegaly, cardiomyopathy, and skeletal dysplasias and deformations [1][2][3][5]. There is no report of storage disease status in Iran.

## 2. Objectives

We conducted a retrospective study to evaluate the pathology specimens of subjects who were referred to the largest tertiary pediatric referral center for storage diseases.

## 3. Patients and Methods

This retrospective study was conducted on liver pathology specimens from 2216 subjects between 1996 and 2007. Participants included pediatric patients aged under 16 years who were referred to the largest tertiary pediatric referral center in Iran, with more than 6000 admissions annually. Approximately 1000 patients are admitted our gastroenterology each year. The pathology specimens were stained with hematoxylin and eosin, periodic acid-Schiff (PAS) trichorom, and PAS with diastase. Two expert pathologists evaluated the specimens for histopathological features of storage disease-namely GSD, LSD (Gaucher and Niemann-Pick), and MPS.

Patient data, such as age, sex, family history, and overall clinical features, were also extracted from patients' files. Data were presented as number, percentage, mean, and range. A combination of clinical and laboratory findings was used to assess the final diagnosis. The presence of Kupffer cells with wrinkled cytoplasms, which are prominent in PAS staining, suggested a diagnosis of Gaucher disease. The presence of large foam cells in sinusoids with a negative PAS stain was used as a diagnostic pathological criterion for Niemann-Pick disease. Liver tissue with a swelled cytoplasm that was filled with pink material and closure of sinusoids by plant-like swallowed hepatocytes suggested GSD. GSD Type I and Type III are PAS (+) and sensitive to diastase. Mild fibrosis is usually seen in GSD Type I and severe fibrosis is seen in GSD Type III, but it is difficult to distinguish severe Type I and Type III disease. In fact, immunohistochemical assay and genetic studies are needed to achieve a net diagnosis for liver storage diseases. Large, round hepatocytes with a ground-glass appearance are usually seen in Type IV. Additionally, PAS (+) and diastase-resistant pinkish material that is surrounded by a clear space (fixation artifact causing retraction of the cytoplasm from cell membrane) is a pathogenomonic pathologic feature of Type IV GSD. This study was approved by the ethical committee of Tehran University of Medical Sciences and conforms to the provisions of the Declaration of Helsinki (as revised in Edinburgh 2000). Although this study was retrospective and the data were extracted from the patients' files at the time of biopsy, a consent form was obtained from the patients' parents.

## 4. Results

Of the 2216 specimens that were evaluated for storage diseases, 117 had evidence of storage diseases. Seventy patients were males and 47 were females. Mean age was 32.8 months. There was no case aged under 1 month. GSD was seen in 85 cases, LSD in 31 patients, and MPS in 1 case. LSD was more prevalent in those aged between 1 month and 1 year, whereas GSD was seen more often in those aged between 1 and 6 years. Most cases were aged between 1 and 6 years. Most patients with LSD and GSD had unknown types of the disease. The most common known types in the LSD and GSD groups were Niemann-Pick disease and GSD type I respectively. Detailed information on the distribution is presented in Table 1.

**Table 1:** Age and Gender Distribution of Liver Storage Disease

	**Total, No. (n = 117)**	**Age, mo**			**Gender**	
		**1 – 12**	**12 - 72**	**> 72**	**Male (n = 70)**	**Female (n = 47)**
GSD a	85	17	58	10	-	-
Type I	18	4	13	1	13	5
Type IV	3	1	2	-	1	2
Unknown	64	12	43	5	32	32
LSD a	31	15	12	4	-	-
Gaucher’s disease	8	3	3	2	7	1
Niemann Pick’s	11	7	4	-	8	3
Unknown	12	5	5	2	8	4
MPS a	1	-	1	-	1	-

^a^ Abbreviations: GSD, glycogen storage disease; LSD, lysosomal storage disease; MPS, mucopolysaccharidoses

Seven patients (6%) suffering from storage disease had a positive family history for such diseases. 99 patients had negative family histories. Unfortunately the family histories for 11 patients were unavailable. The most common manifestation was hepatomegaly, which was present in 108 (92.3%) patients; 39.3% of patients had splenomegaly, 9.4% had seizure attacks, 5.1% had jaundice, and 5.1% had abnormal faces. Laboratory abnormalities were also prevalent in the patients. The most common laboratory abnormality was elevated transaminase levels, present in 90 (76.9%) patients. Only 1 patient had normal aminotransferase levels; 26 had an unknown liver enzyme status. Hypoglycemia was present in 23 cases with GSD and no patients with LSD. Other abnormal findings are presented in Table 2.

**Table 2:** Manifestations of Storage Diseases (n = 117)

**Manifestation **	**Frequency, %**
Hepatomegaly	92.3
Splenomegaly	39.3
Elevated aminotranferase	76.9
Elevated triglyceride or cholesterol	35
Abnormal coagulation profile	5.1
Jaundice	5.1
Seizure	9.4
Abnormal facial features	5.1
Metabolic acidosis	10.3
Hypoglycemia	19.7
Others a	29

^a^ Strabismus, cloudy cornea, pruritus, spider angioma, palmar erythema, fever and sweating, anemia, vomiting, respiratory distress, chronic diarrhea, kidney problem, abdominal collateral vessels, ascites, cirrhosis, severe headache, myotonia

## 5. Discussion

The purpose of this study was to evaluate liver pathology specimens of 2216 children who were referred to a children's medical center for evidence of liver storage diseases. We found that 117 of them had storage diseases, of which GSD was the most common. Hepatomegaly and abnormal liver enzymes were the most common clinical and laboratory findings, respectively. Most studies evaluate storage diseases specifically; i.e., based on their type. Most of our patients had GSD. Although studies on the prevalence of GSD (in general) are lacking, evidence on the prevalence of each type of GSD and LSD supports the finding of our study that GSD is generally more common than LSD [8]. Our study noted a lower prevalence of Gaucher disease compared with Niemann-Pick disease, which contrasts the finding of studies in the Netherlands [7] and Australia but similar to those in Portugal and the Czech Republic [8][9]. For example, in a study by Poupetova et al. in the Czech Republic, a total of 478 patients with LSD were identified, with lipoidosis, such as Niemann-Pick and Gaucher, as the most prevalent types [8]. We only found 1 case with MPS, whereas they found 119 cases-25% of their sample. Although our patients were enrolled from all around our country, our study was not a prevalence study, and the findings can not be interpreted as those from a population based study.

Hepatomegaly was the most common finding in our patients, which is consistent with the high rate of liver involvement in both LSDs and GSDs. However, liver biopsies may bias this finding, because the liver must have been involved to have been included in our study. Other manifestations, including neurologicall abnormalities and dysmorphic features, were also present in our patients, which is consistent with many studies [1][2][4][5][8][10][11]. The laboratory findings of our study are consistent with several others, and it is not surprising that hypoglycemia was not observed in LSD. Again, increased liver enzymes were the most prevalent laboratory finding. Other studies, however, separated the laboratory features of different kinds of storage diseases, based on their type. For example, in GSD type Ia, hypoglycemia and acidosis are the most striking features, rather than abnormal liver enzymes and features of liver failure, such as jaundice. In GSD type IV, abnormal transaminase is a prominent feature, along with hepatomegaly [10][12]. As another example, Gaucher disease primarily involves bone and bone marrow, thus leading to cytopenia [13]. The most important advantage of our study is its large sample size-recruited from the largest tertiary pediatric referral center. The other advantage was that our study was the first to address storage disease overall in Iran.

Our study had several limitations; first, the design of the study was of a retrospective nature, which may potentially have caused recall bias and missing data. Second, immunohisctochemical assay and genetic studies are the mainstays of the diagnosis of storage diseases, unfortunately the design of the study based on the histopathologic diagnosis of the disease due to lack of the required technologies at the time of assessment, prevented the study from having high diagnostic value, particularly for those types of GSD that cannot be distinguished based on histopathological features. Third, the thesis on which the paper is based did not separate clinical presentation and laboratory studies based on the type of storage disease.

In conclusion, despite its retrospective nature and limitations, our study is the first to address storage diseases in Iran. GSD is the most prevalent storage disease, and hepatomegaly and abnormal liver enzymes are the most common abnormal findings. Further studies with more precise data that are preferably prospective in nature are required to give us a full picture of storage diseases in Iran.
